# Optimal Conditions for In Vitro Assembly of Respiratory Syncytial Virus Nucleocapsid-like Particles

**DOI:** 10.3390/v15020344

**Published:** 2023-01-25

**Authors:** Yunrong Gao, Anirudh Raghavan, Bowei Deng, Jae Lee, Bo Liang

**Affiliations:** Department of Biochemistry, Emory University School of Medicine, Atlanta, GA 30322, USA

**Keywords:** respiratory syncytial virus (RSV), nucleocapsid (NC), nucleocapsid-like particles (NCLPs), salt concentration, ionic strength, pH, negative stain EM

## Abstract

The nucleocapsids (NCs) of the respiratory syncytial virus (RSV) can display multiple morphologies in vivo, including spherical, asymmetric, and filamentous conformations. Obtaining homogeneous ring-like oligomers in vitro is significant since they structurally represent one turn of the characteristic RSV NC helical filament. Here, we analyzed and optimized conditions for forming homogenous, recombinant nucleocapsid-like particles (NCLPs) of RSV in vitro. We examined the effects of modifying the integrated RNA length and sequence, altering incubation time, and varying buffer parameters, including salt concentration and pH, on ring-like NCLPs assembly using negative stain electron microscopy (EM) imaging. We showed that high-quality, homogeneous particles are assembled when incubating short, adenine-rich RNA sequences with RNA-free N associated with P (N^0^P). Further, we reported that a co-incubation duration greater than 3 days, a NaCl concentration between 100 mM and 200 mM, and a pH between 7 and 8 are optimal for N-RNA ring assembly with polyadenine RNA sequences. We believe assembling high-quality, homogeneous NCLPs in vitro will allow for further analysis of RSV RNA synthesis. This work may also lend insights into obtaining high-resolution nucleocapsid homogeneous structures for in vitro analysis of antiviral drug candidates against RSV and related viruses.

## 1. Introduction

Respiratory syncytial virus (RSV) is a significant viral pathogen most commonly responsible for inducing lower respiratory tract diseases in infants, older adults, and immunocompromised individuals globally [1,2,3,4]. In infants, RSV primarily causes bronchiolitis and pneumonia [5], and pathology manifests more generally as immunological hypersensitivity in the respiratory tract associated with increased vulnerability to the development of allergy and asthma [6,7]. Currently, there are no approved vaccines for immunization against RSV for the pediatric or elderly population. RSV is classified as an enveloped nonsegmented negative sense (NNS) RNA virus of the *Pneumoviridae* family [8]. The genome of a commonly studied RSV A2 strain comprises 15,222 nucleotides and contains ten sequential genes that encode eleven different proteins (of which nine are structural proteins and two are non-structural proteins) [9]. From 3’ to 5’, the genes encode the following proteins: non-structural proteins 1 and 2 (NS1 and NS2), the nucleoprotein (N), the phosphoprotein (P), the matrix protein (M), the small hydrophobic protein (SH), the glycoprotein (G), the fusion protein (F), M2-1 and M2-2, and a large protein (L, an RNA-dependent RNA polymerase) [10]. Each gene is situated between gene start (GS) and gene end (GE) sequences. The RSV genome also contains two extragenic regions: a 44 nucleotide (nt) leader (Le) sequence and a 155 nt trailer (Tr) sequence at the 3’ and 5’ ends, respectively, and the Le sequence functions as a single promoter for both transcription and replication. During viral replication, the RSV antigenome (genome complementary sequence) uses the Tr complementary (TrC) sequence as the replication promoter [11].

The RSV N protein consists of 391 residues and has a molecular weight (M.W.) of about 43 kDa [12,13]. The RSV genomic RNA is encapsidated with the N protein to form a helical nucleocapsid (NC), which is then used as a template for RNA synthesis by the RSV polymerase (a complex of L:P proteins) [14]. The RSV P protein is a multi-functional protein and functions as a co-factor for RSV L and as a chaperone for keeping N free of RNA, thereby affecting the efficiency of NC assembly and modulating the activity of the RNA-dependent RNA polymerase [15,16]. P protein can interact with RNA-free N protein (N^0^) with its N-terminal domain (P_NTD_) and with the NC with its C-terminal domain (P_CTD_) [17,18]. Prior research has shown that overexpressed recombinant N protein could bind with random cellular RNA to form nucleocapsid-like particles (NCLPs) without sequence specificity [14]. The atomic resolution crystal structure of the RSV ring-like NCLPs depicted that each N subunit coordinated with 7 ribonucleotides and is assembled as a core region, with a distinct N-terminal domain (NTD) and C-terminal domain (CTD), each having its respective extensions termed the N-arm and the C-arm [14]. Certain key residues of RSV N interact with either ribose C2 hydroxide moiety or the phosphate backbone of the encapsidated RNA via their main chain or side chain [14]. Positively charged residues, including K170, R184, and R185, and uncharged polar residues, including N188, Y337, S313, and T315, interact with the phosphate backbone of the RNA sequence [14]. Residues A172, V256, G335, and R338 interact with the ribose C2 hydroxide via their main chain [14]. These interactions stabilize RNA within the RNA-binding groove of RSV N. The N-arm and C-arm serve as linkers between subunits in the RNA-bound ring by forming stabilizing interactions with adjacent subunits in the NCLPs at the 5’ and 3’ ends, respectively [14]. The RNA binds to a groove formed at the interface between the N- and C-terminal domains [14]. A prior lower-resolution electron microscopy (EM) helical reconstruction of the RSV NCLPs has previously characterized it as a flexible, left-handed, helical structure with a pitch of 68 Angstroms (Å) [9]. With the application of cryoEM, a series of NCLPs structures in the families of *Paramyxoviridae* (e.g., measles virus) [19], *Orthomyxoviridae* (e.g., Influenza virus) [20,21], *Filoviridae* (e.g., Ebola virus) [22], and *Bunyaviridae* (e.g., Hantavirus) [23] have been determined.

Despite the successful reconstitution of the RSV polymerase transcription assay in vitro, the templates used were naked RNA sequences [24]. Additionally, the mechanism of nucleocapsid disassembly and reassembly during the process of RNA synthesis remains to be fully elucidated. Reconstructing high-quality and homogeneous NC particles presents a significant challenge because overexpressed N protein can non-specifically bind to random template RNAs, mostly cellular RNAs, instead of RSV-specific genomic RNA [9,25]. We have previously demonstrated that P_NTD_ can be exploited as a chaperone to block N association with non-specific RNAs via the co-expression of P_NTD_ and N, thus successfully obtaining RNA-free N^0^ in RSV on a large scale and in a trackable manner [25]. We have also previously reported that efficient and stable NCLPs assembly in RSV with purified RNA-free N requires RNA that is at least 7 nt in length and that NC assembly can vary further depending on the length and specific sequence of the RNA encapsidated [25]. However, the constructed NCLPs lacked homogeneity, raising the need to optimize assembly conditions. The existing in vitro RSV RNA-dependent RNA polymerase (RdRp) assays use naked RNAs as templates [24,26]. In contrast, the RNA is fully encapsidated by N in vivo. Thus, the capability of preventing non-specific N encapsidation of RNA and modifying parameters to control the assembly of the N-RNA complex in vitro may provide advantages for developing high-throughput assays to study RNA synthesis and analyze antiviral inhibition of the N-RNA complex.

This study aimed to successfully reconstitute RNA-free nucleoprotein (N^0^) with P_NTD_ (P_1-126_) for well-defined, homogeneous N-RNA construction in vitro with short, specific RNA sequences. NC morphologies in vitro were qualitatively distinguished between using negative-stain EM imaging. We demonstrated that the optimal construction of homogeneous NCLPs depends upon multiple variables, including incubation time, length, the sequence of the encapsidated RNA, buffer salt concentration, and buffer pH. We demonstrated that the efficient assembly of homogeneous RSV N-RNA ring particles in vitro is favored by co-incubating adenine-rich RNA sequences greater than 6 nt in length with RNA-free N^0^P_NTD_ for greater than 3 days, a buffer NaCl concentration between 200 and 400 mM, and a pH value of between 7 and 8. We believe that the formation of stable, homogeneous NCLPs in vitro can be further used to construct a high-resolution structure through cryo-EM and serve as a platform for exploring both of the mechanisms involved in RSV RNA synthesis and elucidating methods for antagonizing the NC complex for therapeutic benefit.

## 2. Materials and Methods

### 2.1. N^0^-P_1-126_ Protein Expression and Purification

The N^0^-P_1-126_ plasmid was synthesized by SynbioTechnologies (NJ, USA), which allowed for the expression of the N^0^-TEV-P_1-126_-10xHis fusion protein. The *E. coli* BL21 (DE3) chemically competent strain was utilized for producing N^0^-P_1-126_. Cells were cultured at 37 °C in Miller’s Luria Broth medium until the A_600_ absorbance value reached 0.8. Expression was induced after lowering the temperature to 16 °C and incubating with 0.5 mM of isopropyl β-d-1-thiogalactopyranoside (IPTG) overnight. Cell lysis was conducted through sonication in lysis buffer A (50 mM of sodium phosphate at pH 7.4, 500 mM of NaCl, 5 mM of imidazole, 10% glycerol, and 0.2% NP-40 detergent) and subsequent centrifugation. The supernatant was then loaded onto a cobalt column equilibrated with lysis buffer. The cobalt column (GoldBio, St Louis, MO, USA) was washed with buffer B (50 mM of Tris-HCl at pH 7.4, 1 M of NaCl, 10% glycerol, and 5 mM of imidazole) and buffer C (50 mM of Tris-HCl at pH 7.4, 500 mM of NaCl, 10% glycerol, and 5 mM of imidazole). The protein was eluted from the cobalt column with buffer D (50 mM of Tris-HCl at pH 7.4, 500 mM of NaCl, and 400 mM of imidazole). Further protein purification was performed via Q column (HiTrap Capto Q ImpRes, 5 × 5 mL, Cytiva, Marlborough, MA, USA), followed by TEV (tobacco etch virus) cleavage with dialyzation against buffer E (25 mM of Tris-HCl at pH 8.0, 300 mM of NaCl, 10% glycerol, 1 mM of DTT). The TEV enzyme was removed by re-running the Q column (HiTrap Capto Q ImpRes, 5 × 5 mL, Cytiva, Marlborough, MA, USA). Finally, purification was completed using a Superdex 200 gel-filtration column equilibrated with buffer F (20 mM of HEPES at pH 7.4 and 200 mM of NaCl). The purified protein N^0^-P_1-126_ complex was concentrated using a centrifugal concentrator (MilliporeSigma, Burlington, MA, USA) with a molecular weight cut-off (MWCO) of 10 kDa.

### 2.2. In Vitro Assembly of N-RNA Complex

RNA oligos were purchased from Integrated DNA Technologies and saved as stock at a concentration of 100 µM. In order to assemble the NCLPs in vitro, the protein N^0^-P_1-126_ complex and RNA were co-incubated with a molar ratio of 1:1.5. The sample was used directly to make the negative stain grids for screening.

### 2.3. RNase A Digestion

For RNase A (MilliporeSigma, Burlington, MA, USA) treatment, the N^0^-P_1-126_ complex was preincubated in the presence of RNA at 4 °C overnight, then incubated for one hour at room temperature in the presence of RNase A (final concentration 100 µg/mL).

### 2.4. Negative Stain Electron Microscopy Imaging of N-RNA Complexes

First, 3 µL of each of the samples was applied to the clean side of freshly glow-discharged grids and subsequently stained with 1% (*w*/*v*) uranyl formate. Negative stain EM imaging was performed using an FEI Talos L120C electron microscope (Thermo Fisher Scientific, Waltham, MA, USA) operating at 120 keV and equipped with an FEI Ceta 4k × 4k charge-coupled device camera. Micrographs were collected at nominal magnifications of 73,000 (1.97 Å/pixel). The images were acquired at a defocus value of −1.2~−2.0 µm and electron doses of −25 e^−^/Å^2^.

## 3. Results

In this study, we describe the effects of varying sample incubation time, buffer salt concentration, buffer pH, and the length and sequence of encapsidated RNA on in vitro RSV NCLPs assembly. Further, we show an RNase A digestion analysis of NCLPs assembled with varying lengths of short RNA sequences to assess N-RNA complex stability qualitatively.

**Figure 1:**
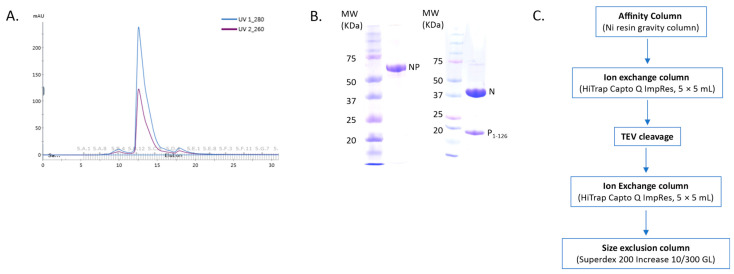
Purification of the recombinant NP protein complex. (**A**) The gel filtration profile of the N^0^P_1-126_ protein complex. (**B**) Protein samples were analyzed by SDS-PAGE. The left panel depicts the fused NP protein sample, and the right panel depicts the sample corresponding to the major gel filtration peak, which is the N^0^P_1-126_ protein complex. (**C**) Outline of the NP protein complex purification.

NCLPs were assembled by incubating RNA-free N (N^0^) with a fixed molar ratio of RNA. For these complexes, a fusion protein comprised of full-length N (N_1-391_) and the N-terminal domain of P (P_1-126_) was utilized, separated by a TEV cleavage domain with a C-terminal 10-His tag. A fixed molar ratio of protein-to-RNA was utilized in this study to achieve saturation of N with RNA when assembling the NCLPs, and protein concentrations were held constant between samples to allow for qualitative comparison. The peak corresponding to the NP_1-126_ complex in the gel filtration profile showed an A_260nm_/A_280nm_ ratio of less than 1, implying no RNA contamination in the sample (Figure 1A,B). A brief overview of the purification protocol to generate the N^0^P_1-126_ complex has been outlined (Figure 1C). We generally observed baseline NCLPs assembly when N and RSV-specific RNA molecules were co-incubated, which reinforced the notion that the polymerization of RSV N and RSV-specific RNA into the ring-like structure is favored and stabilizing. NCLPs were assembled by incubating RNA-free N (N^0^) with a fixed molar ratio of RNA. Negative stain EM imaging was used to analyze the NCLPs ring assembly. To qualitatively describe the particle quality, we use the term “efficiency”, referring to the proportion of particles within a sample that resemble a complete ring-like N-RNA oligomeric assembly. To qualitatively define the energetic favorability of oligomerization, we use the term “propensity”, referring to the raw proportion of the specific type of oligomeric structure observed within the sample, including complete N-RNA ring assemblies, incomplete N-RNA ring assemblies, and particle aggregates.

### 3.1. Within 7 nt RNA Sequences, Adenine Is the Preferred Ribonucleotide for Efficient N-RNA Ring Assembly

**Figure 2:**
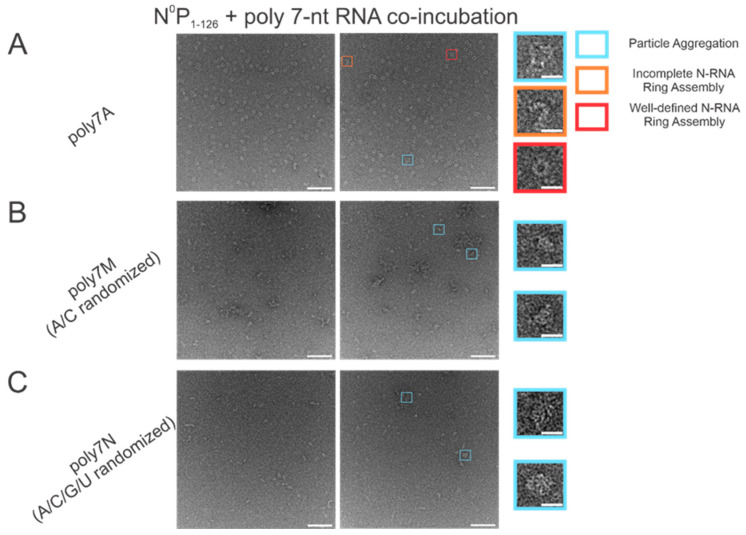
The poly7A sequence was preferred for N-RNA ring assembly over randomized 7 nt sequences with either an A and C or A/C/G/U randomized sequence. Two representative negative stain images are included for a statistically representative depiction. (**A**) poly7A; (**B**) poly7M (adenine/cytosine randomized sequence); (**C**) poly7N (adenine/cytosine/guanine/uracil randomized sequence). Markers have been utilized to denote representative particle morphologies in A. Orange rectangles denote incomplete ring assemblies, blue rectangles denote particle aggregation, and red rectangles denote complete ring assemblies. Scale bars within the original images correspond to 100 nm, and scale bars within the enlarged images correspond to 20 nm.

The atomic resolution crystal structure of the RSV ring-like NCLPs showed that each N subunit coordinates with 7 ribonucleotides and assembles as a core region [14]. In comparing 7 nt polyadenine (polyA) sequences with randomized sequences consisting of either adenine (A) and cytosine (C) (Figure 2B) or an A/C/G/U randomized sequence (Figure 2C), complete N-RNA ring NCLPs were only obtained with the poly7A sequence (Figure 2A). When N^0^P was incubated with the poly7N RNA sequence that contained a random 7 nt sequence of the 4 ribonucleotides, only particle aggregates could be observed; distinct ring-like structures could not be observed from negative-stain EM imaging (Figure 2C). Further, more particle aggregation was observed with the A/C randomized sequence than the A/C/G/U randomized sequence, suggesting that with 7 nt RNA sequences, adenine increased the propensity for N-RNA ring assembly and added stability favoring the efficient formation of higher-order morphological structures.

### 3.2. N-RNA Ring Assembly Propensity Increases when Encapsulating polyA RNA over 6 Ribonucleotides in Length

**Figure 3:**
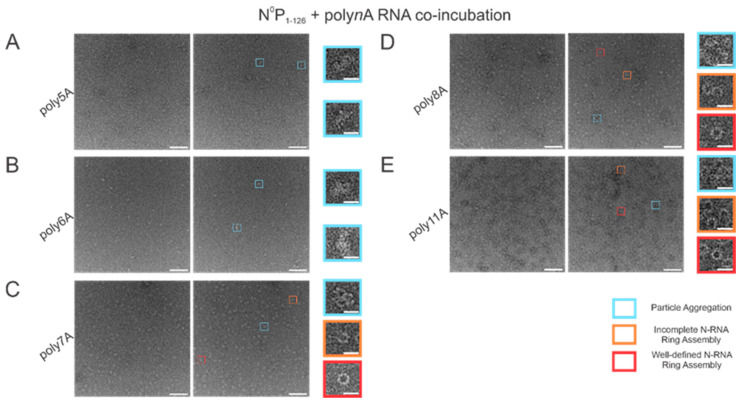
Propensity for N-RNA particle assembly increases when using polyadenine sequences greater than 6 nucleotides in length. Two representative negative stain images are included for a statistically representative depiction. (**A**) poly5A; (**B**) poly6A; (**C**) poly7A; (**D**) poly8A; (**E**) poly11A. Markers have been used to denote representative particle morphologies. Orange rectangles denote incomplete ring assemblies, blue rectangles denote particle aggregation, and red rectangles denote complete ring assemblies. Scale bars within the original images correspond to 100 nm, and scale bars within the enlarged images correspond to 20 nm.

With varying lengths of polyadenine sequences, we observed that the minimal length required for well-defined N-RNA ring assembly was 7 since no particles were observed for poly5A or poly6A (Figure 3A,B). An increased propensity for N-RNA oligomerization was observed when using poly7A; at this length, the first well-defined N-RNA rings were seen (Figure 3C). However, when N^0^P was incubated with poly8A, NCLPs ring formation propensity seemed to have increased even further at the detriment of efficiency, leading to a greater proportion of incomplete ring assemblies and particle aggregates but a greater total density of the particles captured (Figure 3D). Images of N-RNA with the poly11A sequence depicted a high density of the well-defined N-RNA ring structures, suggesting that there was now a high-efficiency assembly of N-RNA rings without detriment to the ring formation propensity (Figure 3E). These findings highlight the requirement of a minimum sufficient polyadenine sequence length such that N-RNA ring assembly efficiency may be balanced with the propensity for oligomerization.

### 3.3. Increasing Incubation Time Increases the Proportion of Complete RSV NCLPs Assemblies with poly7A RNA

**Figure 4:**
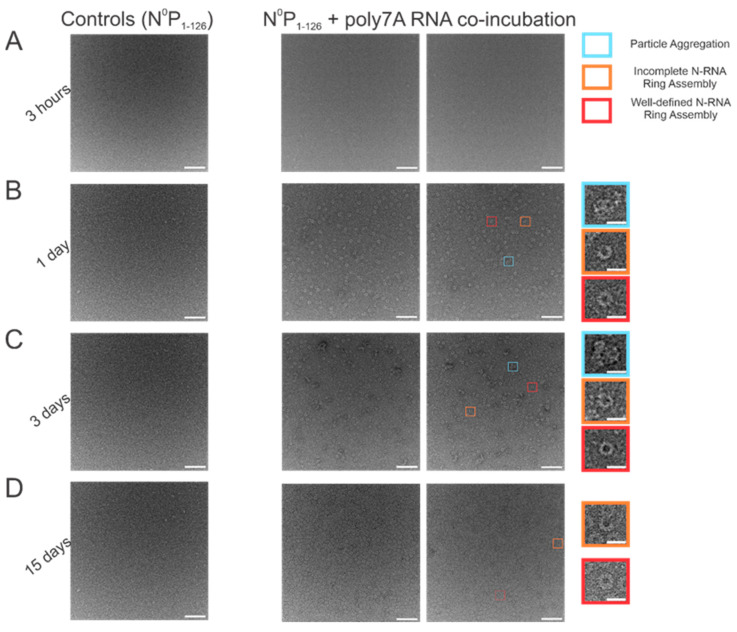
Increasing incubation time increases both the propensity to form RSV N-RNA rings in vitro and assembly efficiency. (**A**) 1 h incubation period; (**B**) 1-day incubation period; (**C**) 3-day incubation period; (**D**) 15-day incubation period. The first image in each column represents negative controls (N^0^P_1-126_ incubated without RNA), followed by two representative negative stain images. Markers have been added to the figure series to denote distinct and representative particle morphologies. Orange rectangles denote incomplete ring assemblies, blue rectangles denote particle aggregation, and red rectangles denote complete ring assemblies. Negative controls depict that RNA is essential for RSV N oligomerization and that the N^0^P protein complex cannot independently form highly ordered ring particles. Scale bars within the original images correspond to 100 nm, and scale bars within the enlarged images correspond to 20 nm.

When N^0^P was incubated with poly7A RNA, and the particle sets were analyzed, a greater number of closed-ring particles were identified when the samples were incubated for longer periods at 4 °C. Longer incubation times served to generate a larger number of closed-ring N-RNA assemblies with defined shapes. At the 1 h time point, no appreciable NCLPs formation was observed (Figure 4A). With incubation of the sample overnight (Figure 4B), an observable proportion of particles captured consisted of unstable, incomplete rings. This proportion decreased with increasing incubation time, while ring formation propensity increased (Figure 4C,D). At the 3-day incubation point, a high proportion of particle aggregation was observed, seemingly due to the increased propensity but subpar assembly efficiency (Figure 4C). At the 15-day incubation point, ring formation efficiency was the highest, with minimal particle aggregation, incomplete N-RNA ring assembly, and a high proportion of well-defined, complete N-RNA rings.

### 3.4. Increasing Buffer Salt Concentration/ionic strength Tones RSV N-RNA Ring Formation Propensity

**Figure 5:**
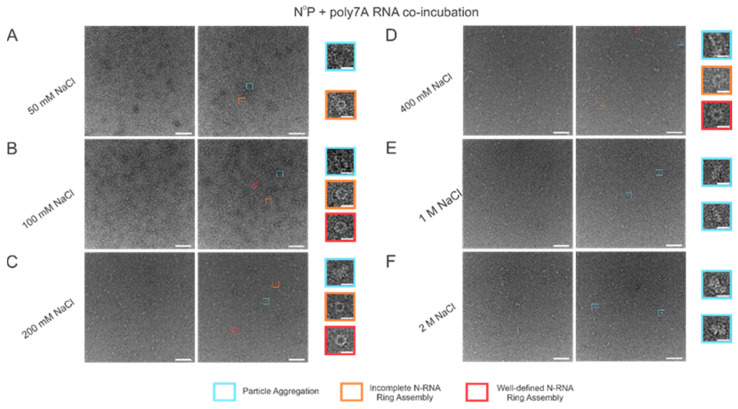
Increasing buffer NaCl concentration decreases N-RNA ring formation propensity while not significantly impacting ring formation efficiency. (**A**) 50 mM of NaCl; (**B**) 100 mM of NaCl; (**C**) 200 mM of NaCl; (**D**) 400 mM of NaCl; (**E**) 1 M of NaCl; (**F**) 2 M of NaCl. Markers have been utilized to denote representative particle morphologies. Orange rectangles denote incomplete ring assemblies, blue rectangles denote particle aggregation, and red rectangles denote complete ring assemblies. Scale bars within the original images correspond to 100 nm, and scale bars within the enlarged images correspond to 20 nm.

When the incubation buffer NaCl concentration was stepped between values starting from 50 mM and extending to 2 M, a significant alteration in RSV N-RNA ring formation propensity was observed, with seemingly no qualitative change in ring formation efficiency. At 50 mM of NaCl, ring formation propensity seemed excessively high, leading to widespread aggregation and a general inability to distinguish distinct particle morphologies (Figure 5A). Doubling the salt concentration to 100 mM reduced the degree of particle aggregation observed, and complete and incomplete N-RNA particles could now be identified (Figure 5B). Doubling NaCl concentration again to 200 mM reduced particle aggregation further, and particle morphologies remained observable (Figure 5C). Raising the salt concentration to 400 mM resulted in minimal to negligible aggregation and a further reduction in N-RNA ring formation propensity, reflected by the relatively lower density of particles (Figure 5D). No ring formation was observed at NaCl concentrations of 1 M and 2 M (Figure 5E,F).

### 3.5. N-RNA Ring Assembly Propensity Is Highly Sensitive to pH

**Figure 6:**
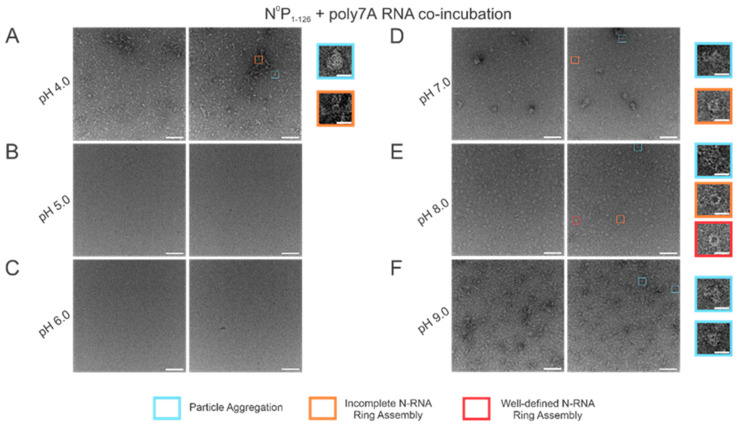
N-RNA particle assembly efficiency is in a narrow pH band around pH 8. Two representative negative stain images are included for a statistically representative depiction. (**A**) pH 4; (**B**) pH 5; (**C**) pH 6; (**D**) pH 7; (**E**) pH 8; (**F**) pH 9. Markers denote representative particle morphologies. Orange rectangles denote incomplete ring assemblies; blue rectangles denote particle aggregation; red rectangles denote complete ring assemblies. Scale bars within the original images correspond to 100 nm, and scale bars within the enlarged images correspond to 20 nm.

Using high RNA:N ratio samples, we observed marked particle aggregation with virtually no well-defined ring formation at both extremes of the pH range (4–9) tested (Figure 6A,F). Notably, initial incremental pH changes appeared to alter N-RNA ring formation propensity drastically: increasing pH from 4 to 5–6 eliminated particle formation altogether, suggesting that N and RNA remained isolated (Figure 6B,C). At pH 7, N-RNA particles were observed, albeit in aggregates, but the propensity for ring formation is moderate relative to pH 4 and pH 9 (Figure 6D). Well-defined N-RNA rings with high efficiency and moderate propensity were observed only at pH 8 (Figure 6E), and mass aggregation was observed at pH 9 (Figure 6F). Due to the more gradual morphological transition between pH 7 and 8, the optimal pH for high N-RNA ring formation efficiency likely hovers around 8. This relatively narrow pH range suggests that ring assembly is susceptible to pH changes.

### 3.6. RNase A Treatment of N-RNA Complexes Depicts That RSV NCLPs Assembled In Vitro Are Stable

**Figure 7:**
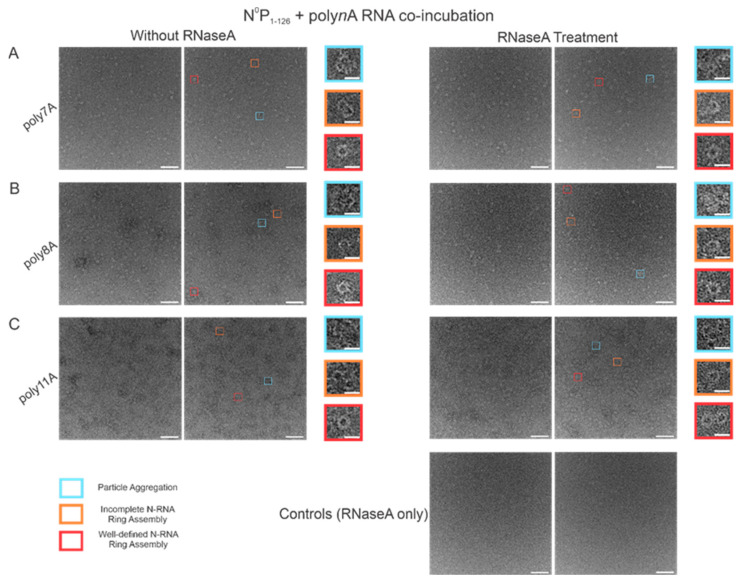
RNase A treatment significantly reduced the sample proportion of complete and incomplete N-RNA rings and particle aggregates. Two representative negative stain images are included for a statistically representative depiction. (**A**) poly7A; (**B**) poly8A; (**C**) poly11A. Orange rectangles denote incomplete ring assemblies; blue rectangles denote particle aggregation; red rectangles denote complete ring assemblies. Scale bars within the original images correspond to 100 nm, and scale bars within the enlarged images correspond to 20 nm.

In order to test the stability of the RSV NCLPs assembled in vitro, RNase A was used to digest the RNA in the NCLPs with RNA sequence lengths greater than 7 nt, reducing the sample proportion of all observed oligomer types. For the 7 nt, 8 nt, and 11 nt sequences tested, RNase A treatment of the N-RNA complexes resulted in a reduction in the sample proportion of incomplete ring oligomers and particle aggregates (Figure 7). A significant reduction in the proportion of complete, well-defined N-RNA rings was only noted with RNase treatment of the N-RNA complexes that contained poly8A (Figure 7B) and poly11A (Figure 7C).

## 4. Discussion

**Figure 8:**
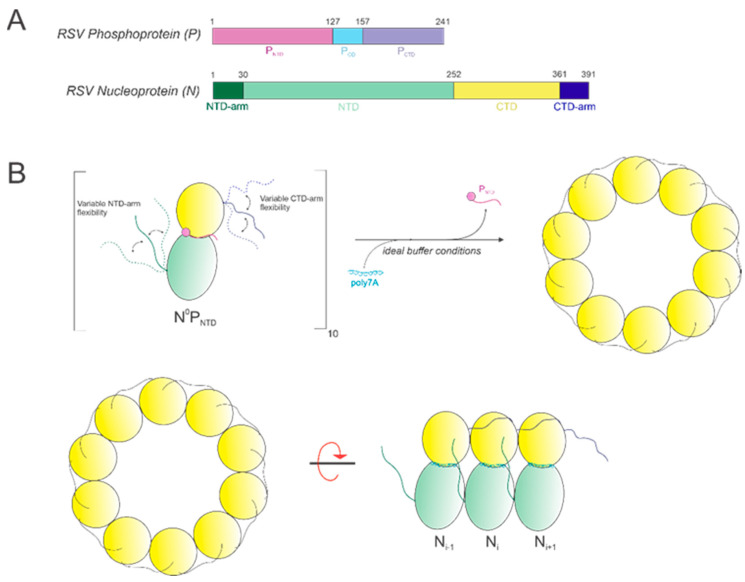
(**A**) Schematics of the RSV phosphoprotein (P) and nucleoprotein (N). (**B**) Model depicting oligomerization of the base unit N^0^P_NTD_ with poly7A under optimal conditions. N-monomers oligomerize into a decameric ring, where P is (i) a molecular chaperone of N to produce the RNA-free N^0^P complex and (ii) is substituted with the co-incubated RNA sequence to yield the N-RNA ring.

In this study, we show that it is possible to construct high-quality, homogeneous NCLPs assemblies within certain ranges of select buffer parameters and RNA characteristics, which can further serve as a manipulatable base unit for performing NC functional studies, such as in vitro RNA polymerase assays. An RNA-free NP fusion protein consisting of full-length N and the N-terminal domain of P (Figure 8A) was used as a minimal unit for NCLPs decameric ring assembly (Figure 8B).

Our results agree with our previously published results [25] in that A is the preferred ribonucleotide for both encapsidation by N and subsequent NCLPs assembly. A total of 7 nt sequences were used since this was the minimal length required for successful encapsidation by N in vitro, maintaining consistency with both our previous findings [25] as well as recent results by Gonnin and colleagues [27]. Randomizing between A and just one other ribonucleotide, C, resulted in particle aggregation with no well-perceived N-RNA oligomeric rings. Randomizing between all four ribonucleotides resulted in particle aggregation as well. However, incubating N^0^P with the 7 nt A/C/G/U randomized sequence resulted in a seemingly lowered propensity for particle oligomerization relative to the A and C randomized RNA. This suggests that when using 7 nt RNA sequences, A has a greater binding affinity to RSV N, making N more amenable to RNA encapsidation through certain mechanisms, such as favoring P displacement from N**^0^**.

We also compared N-RNA ring formation propensity and efficiency when incubating N^0^P with short polyA RNA sequences of varying lengths: the first well-defined N-RNA rings were observed when using poly7A RNA. This suggests that shorter RNA sequences may not stabilize the equilibrium from the RNA-free N conformation to the RNA-bound N conformation required for oligomerization. Interestingly, ring formation propensity increases, whereas formation efficiency reduces when incubating N^0^P with poly8A; ring efficiency is restored despite an even greater oligomerization propensity when incubating N^0^P with poly11A. This is potentially suggestive of different N-RNA binding modalities that may depend on encapsidated RNA length. In RSV, when using an RNA length equivalent to the length of RNA bound by each N monomer, which is 7 nt, the encapsidation conformation may follow that of other viruses in the *Mononegavirales* order. For instance, in the Measles virus, each N monomer binds 6 ribonucleotides such that its 15,894 nt genome is completely encapsidated and is nuclease-resistant [28,29]. Alternatively, for sequences longer than 7 nt, the RNA could be overhanging between N monomers, bridging consequent N monomers into a continuous ring, or randomly oriented within the oligomeric ring structure. However, it is also possible that some N monomers remain in the N^0^ conformation within the ring and do not bind RNA. Results from the RNase A assay showed that N-RNA ring assembly propensity with poly7A RNA was impacted with RNase A treatment to a far lesser extent than for either of the longer polyA sequences tested: poly8A and poly11A. There was a reduction in the proportion of all observed morphologies regardless of polyA length, which may indicate that even with one specific RNA length, multiple binding configurations can be adopted depending on energetic favorability. This indicates that longer RNA may form overhangs that can potentially serve as nucleation sites for oligomerization, which are otherwise eliminated with RNase A treatment. However, further work needs to be performed concerning structural analyses of N-RNA interactions at the RNA-binding groove to ascertain any such possibility.

We then modified the incubation time and two buffer parameters: NaCl concentration and pH. While complete N-RNA rings were observable at 1 day, 3 days, and 15 days, the propensity for ring formation was much greater, as was the efficiency of well-defined N-RNA rings. At 15 days, there were minimal particle aggregates, suggesting that thermodynamic stability may have been attained for many N-RNA conformers. Thus, it may be the case that particle aggregates may be a result of kinetically favorable interactions, or perhaps particle aggregates are the predominant particle morphology corresponding to kinetically trapped intermediates. Our data suggest that complete N-RNA rings are the thermodynamically stable conformation for RSV NCLPs.

As elaborated on in an earlier section, RSV P functions in preventing both the self-oligomerization of N^0^ and non-specific binding to random cellular RNAs [30,31], and it has been previously shown that the N-terminal domain of P is sufficient for maintaining the monomeric N^0^ form [11,25]. Interestingly, previous literature has also noted that in human RSV (hRSV), the C-arm of N is directly involved in inhibiting the associative interactions between N and RNA. Specifically, a general mechanism of RNA binding inhibition within the *Mononegavirales* order of viruses was described as involving a dual interaction between the N C-arm and the RNA binding domain as well as the binding of P_NTD_ to N, such that interactions with other N monomers and non-viral specific RNAs are impaired [31]. In altering buffer salt concentration, our results indicated that N-RNA ring assembly was optimal in a specific band between 200 mM and 400 mM of NaCl. It may be the case that a higher ionic strength destabilizes the salt-bridging interactions that pack the C-arm against the core of the N-monomer, favoring concurrent placement of RNA nucleotides within the RNA-binding domain and subsequent C-arm linkage with the subsequent N monomer (which is dependent on the RNA sequence length). However, elevating ionic strength above an optimum value may lead to excessive C-arm flexibility to the extent that limiting its flexibility may be entropically unfavorable despite the stabilization conferred. It may be due to monomeric inactivation through association near the RNA binding groove of its resident N monomer or polymerization via linkage to the consequent nucleoprotein monomer to form N-RNA oligomers. This may explain why at least some particle aggregation is observed at the extremes of the salt concentration tested. In relation to pH, however, while optimal N-RNA ring assembly is reserved within a small band between pH 7 and pH 8, virtually no particle formation propensity is observed between pH 5 and pH 6. N-RNA ring assembly appears to be highly sensitive to pH, implying that a significant interaction required for oligomerization may be hindered at certain pH values. Further work is necessary to elucidate the structural impact of modifying buffer ionic strength and pH on the RNA-binding and oligomerization domains of the N protein.

**Figure 9:**
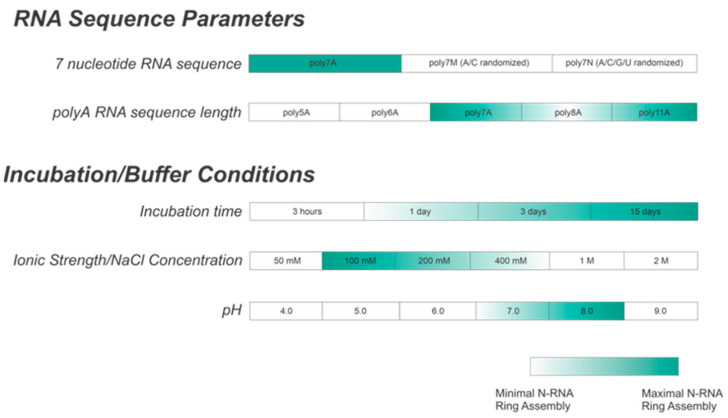
Summary table showing optimal buffer conditions for in vitro N-RNA ring assembly. A is the preferred ribonucleotide for encapsidation. An incubation period of greater than 3 days is preferred, with a buffer NaCl concentration between 100 and 200 mM and a pH between 7 and 8.

While our current study provides a framework of conditions for efficient in vitro N-RNA ring construction (Figure 9), further characterization of in vitro N-RNA genomic structure morphologies that may occur due to interactions with other RSV proteins remains to be conducted. A recent study conducted by Conley et al. [32] revealed that, in cells infected by RSV, N-RNA rings seem to adopt a stacked, helical, filamentous conformation within RSV virions. Additionally, while short RNA sequences were used in our analysis here, further work characterizing conditions for optimal N-RNA ring assembly when using longer sequences of RNA, such as 70 nt RNA, remains to be performed. When considering the in vitro assembly of RSV NCLPs, a noteworthy limitation is that the RNA has already been synthesized before assembling the N**^0^**P complex. However, within infected cells, the N**^0^**P complex assembly occurs concomitantly with RNA synthesis, which may impact NCLPs formation dynamics. Despite this, in vitro studies are still highly informative as they allow for greater NCLPs manipulation, enabling N to encapsidate specific RNA structures/sequences of choice. The ability to construct high-quality NCLPs in vitro and manipulate the RSV RNA synthesis machinery within this environment is, thus, a powerful method that can better inform strategies for constructing other oligomeric structures assumed by the RSV NCLPs within infected cells, such as helical filaments. Optimizing the construction of different NCLPs morphologies will ultimately assist in recreating the native RSV NCLPs structure bound to specific genomic RNA sequences in vitro, which can be used as a model for developing therapeutic interventions against RSV infection.

## Data Availability

Data sharing is not applicable.
